# Emergence of African swine fever in Bangladesh: key findings from an outbreak investigation

**DOI:** 10.1186/s12917-025-04998-0

**Published:** 2025-10-02

**Authors:** Most. Shahana Akter, Md. Nazrul Islam, Md. Riabbel Hossain, Shadia Tasnim, Shukes Chandra Badhy, Md. Golam Azam Chowdhury, Emdadul Haque Chowdhury, Rokshana Parvin

**Affiliations:** 1 Department of Pathology, Faculty of Veterinary Science, Agricultural University, Mymensingh, 2202 Bangladesh; 2 Department of Livestock Services, Ministry of Fisheries and Livestock, Dhaka, Bangladesh; 3 Central Disease Investigation Laboratory (CDIL), Department of Livestock Services, Ministry of Fisheries and Livestock, Dhaka, Bangladesh

**Keywords:** African swine fever, Outbreak investigation, Clinicopathology, Postmortem examination, Bangladesh, Pig

## Abstract

**Background:**

African swine fever (ASF), caused by the African swine fever virus (ASFV), is a significant transboundary animal disease characterized by severe hemorrhages and high mortality in pigs. It poses substantial socioeconomic threats to the global pork industry. This study reports the first in-depth investigation of ASF in Bangladesh, focusing on the clinicopathological observations in naturally infected pigs and the molecular detection of ASFV.

**Case presentation:**

On August 28, 2024, an outbreak occurred on a farm in the Panchagarh district of Bangladesh, which housed 230 indigenous pigs, 210 of which were found dead suddenly. Clinicopathological observations were conducted to assess the tissue changes and confirm the presence of the virus. Postmortem examinations were performed on selected animals and tissue samples, including spleen, lungs and liver, were collected from four pigs for laboratory analysis. Real-time polymerase chain reaction (qPCR) was conducted on the collected samples to detect the presence of ASFV at the molecular level, targeting the B646L gene encoding the p72 capsid protein. Clinically, the pig exhibited high fever (41–42 °C), anorexia, marked dullness, respiratory distress and lethargy. A severe bloody nasal discharge (epistaxis) was also noted, which followed death. Gross pathological findings included red hepatization of the lungs with edema, severe hemorrhagic splenomegaly, and a congested hemorrhagic liver. Histopathological examination of the lungs revealed widespread pneumonia, with predominant infiltration of alveolar macrophages. The spleen exhibited massive multifocal necrosis, while the liver showed significant hepatocellular necrosis, portal hepatitis, macrophage infiltration, and the presence of intracytoplasmic inclusion bodies. All four samples tested positive for ASFV by qPCR.

**Conclusions:**

Since the initial outbreak, no detailed investigation of African swine fever (ASF) has been documented in Bangladesh. This field investigation, therefore, represents the first comprehensive report in the country. Continued in-depth studies, including genomic characterization of the virus and systemic surveillance of the pig population, are essential for the effective control and prevention of ASF in Bangladesh.

## Background

African swine fever (ASF) is the most impactful transboundary animal disease affecting the pork industry. It is a highly contagious disease that causes severe hemorrhages and high mortality rates that may reach 100% within seven days after the onset of clinical signs in both domestic and wild pigs [1]. The first outbreak of ASF occurred in East Africa in the early 1900 s [2]. The disease later spread to Europe and South America during the 1950 s and 1960 s, respectively [3, 4]. Following its spread to various countries, the eradication of ASF required years of coordinated efforts involving surveillance, culling, movement control, and strict biosecurity measures [5, 6]. Unfortunately, after a period of apparent absence, ASF was reintroduced to the Caucasus region of Georgia in 2007, from where it rapidly spread to neighboring countries and beyond [7]. Outbreaks were then reported in different Asian countries between 2018 and 2020 [4]. In Bangladesh, the first confirmed outbreak of ASF was reported on December 21, 2023, at a government-operated pig development farm in the Rangamati district [8]. However, the initial report was limited to outbreak notification, with no published data providing a detailed clinicopathological or molecular investigation of the cases until the present investigation [8].

African swine fever (ASF) is caused by the African swine fever virus (ASFV), a large, double-stranded DNA virus classified under the genus Asfivirus within the family *Asfarviridae* [6]. ASFV exhibits a complex morphology, comprising an icosahedral capsid and an inner lipoprotein envelope that surrounds the central double-stranded DNA core, all enclosed by a thick protein layer [5]. Transmission of ASFV occurs through both direct and indirect contact, including exposure to infected animals, their biological fluids, contaminated fomites, and through bites from infected soft ticks of the *Ornithodoros spp* [9].

The clinical presentation varies depending on the virulence of the virus, the route and load of the infection and the immune status of the host [6]. Common clinical presentations include high fever, anorexia, dullness, depression, vomiting, diarrhea, redness of the skin, staggering gait, lack of fear and breathing difficulty. In the later stages of infection, pronounced hemorrhagic signs such as epistaxis, bloody diarrhea, cutaneous hemorrhage and neurological signs may develop. In case of an acute lethal stage of ASF, most pigs die within 7–14 days post-infection [10, 11].

In case of acute ASF, the common gross findings on post-mortem examinations are congestive-hemorrhagic splenomegaly, hemorrhagic lymphadenitis, pulmonary edema, petechial hemorrhages in the heart, kidney and intestines [1, 6, 12, 13]. The primary targets of the virus are the macrophages and monocytes [13]. Congestive-hemorrhagic splenomegaly with vascular changes, pulmonary edema with pulmonary intravascular macrophages (PIMs), portal hepatitis, and hepatocellular necrosis in the liver are some common histopathological findings [13, 14].

Although ASF was previously reported in Bangladesh, earlier outbreaks had not been thoroughly investigated. Information regarding the circulating viral strains and associated pathomorphological changes remains limited. This report presents a comprehensive investigation of an ASF outbreak in the Panchagarh district of Bangladesh. The study aimed to characterize the clinicopathological manifestations and conduct molecular detection of ASFV in field cases.

## Case presentation

A sudden outbreak of disease was reported on 28 August 2024 at a pig farm in Boda Upazila of Panchagarh, the northernmost district of Bangladesh, located approximately 425 km from the capital, Dhaka. The incident was reported to the Department of Livestock Services (DLS), and a joint investigation was carried out by our team in collaboration with respective DLS personnel. The farm housed 230 indigenous pigs, raised under a scavenging production system. Of these, 210 pigs died suddenly, the remaining were culled following laboratory confirmation of African Swine Fever, according to national disease control protocols to prevent further spread of the virus.

**Fig. 1:**
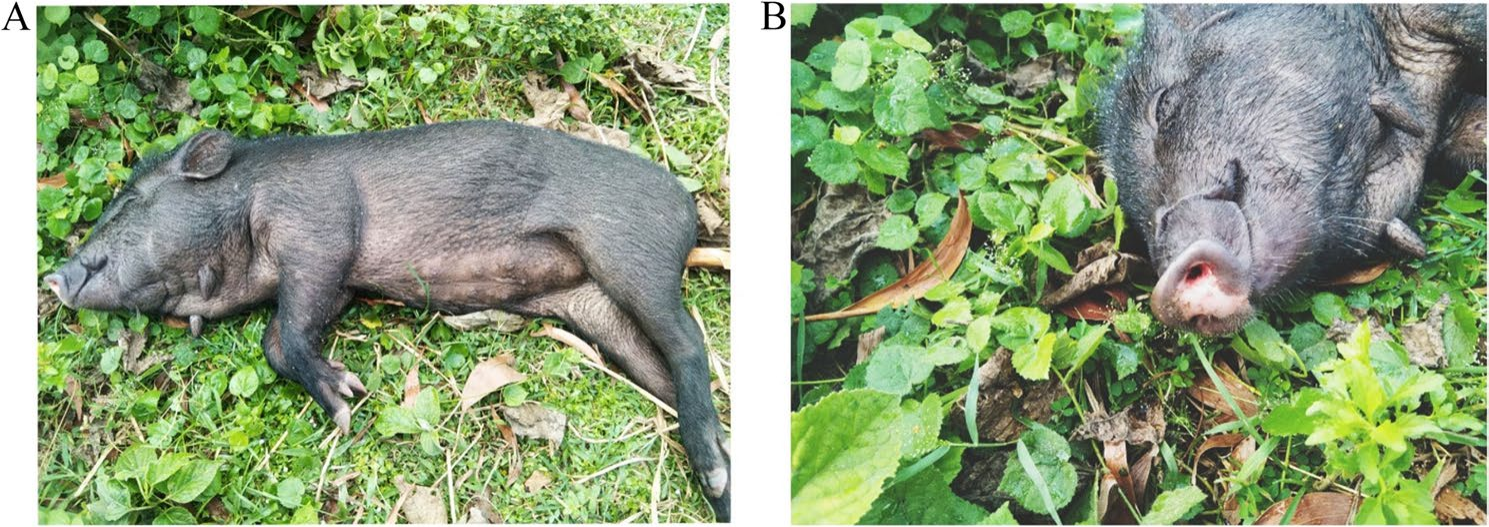
The clinical observation of ASF in indigenous swine. **A** Lethargy associated with high fever and **B** Bloody nasal discharge (Epistaxis)

**Fig. 2:**
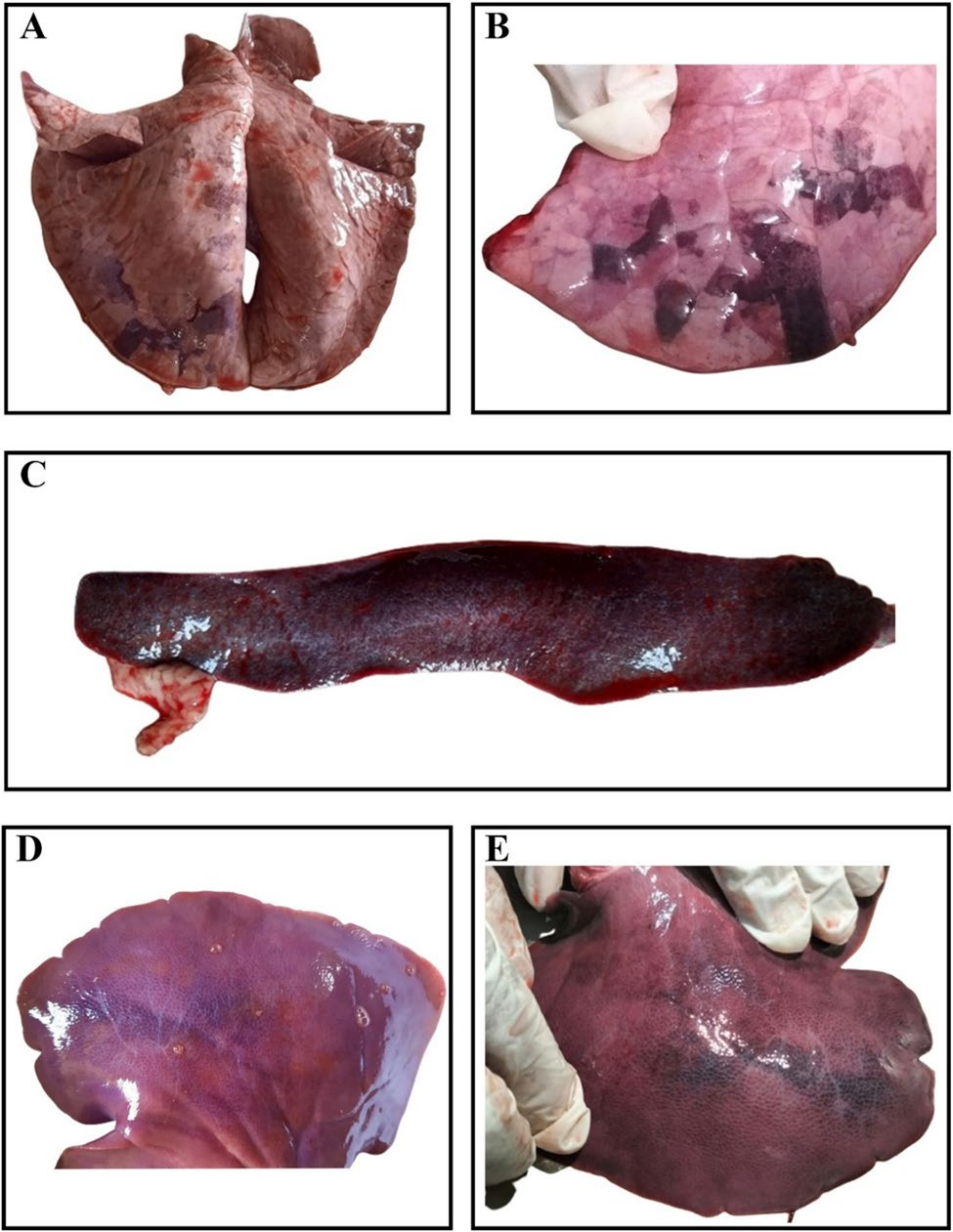
Gross lesions observed in the lungs, spleen and liver. **A**, **B** The lungs show multiple areas of red hepatization across lobes (**C**). Spleen shows massive multifocal necrosis and (**D**-**E**) Liver shows hemorrhage and congestion

**Fig. 3:**
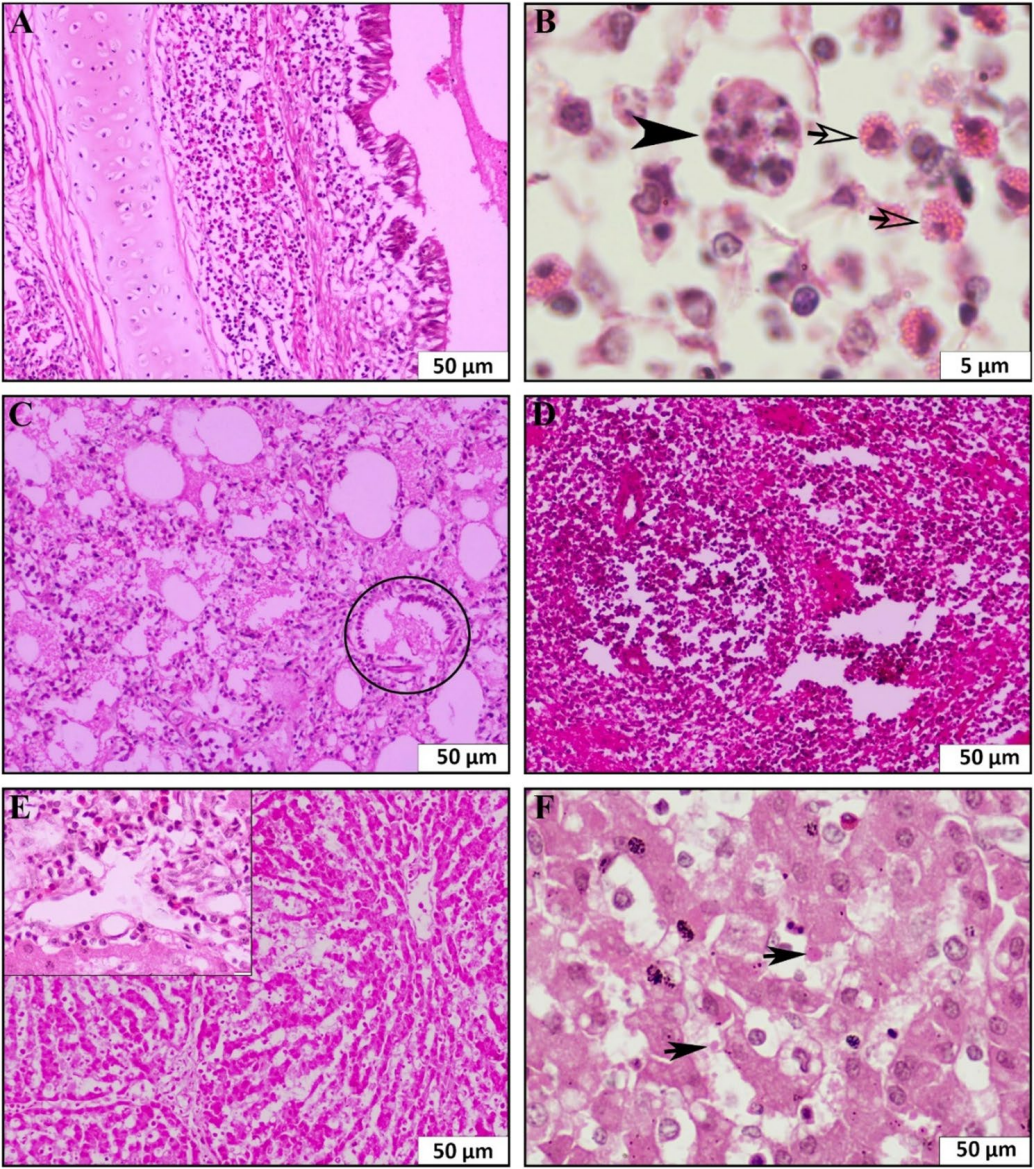
Histopathology of the lungs, spleen, and liver. **A** The lungs show severe bronchitis with marked proliferation of alveolar macrophages and other inflammatory cells. **B **Infected macrophages (arrow) and multinucleated giant cells (arrowhead) are present within the lung parenchyma. **C **Accumulation of proteinaceous matrices, loss of type I pneumocytes, and proliferation of type II pneumocytes arranged in a palisading pattern (black circle). **D** The spleen shows multifocal necrosis with lymphoid depletion. **E** The liver shows widespread hepatocellular necrosis and portal hepatitis (inset). **F** Intracytoplasmic inclusion bodies are visible within the hepatocytes

A systematic outbreak investigation was conducted to assess clinicopathological changes associated with the outbreak and confirm the presence of African swine fever virus (ASFV). The affected indigenous pigs were examined for clinical presentation, as well as gross and histopathological lesions. Clinically, the pigs exhibited marked dullness, anorexia, lethargy, high fever (41–42 °C), epistaxis (Fig. 1), and severe respiratory distress. These ultimately resulted in the death of the pigs. Post-mortem examination was performed with particular attention to the heart, lungs, spleen, liver, intestine, kidneys, and other organs. The lungs showed multiple areas of red hepatization of variable sizes across multiple lobes (Fig. 2A and B), accompanied by edema. The spleen exhibited severe hemorrhagic splenomegaly, characterized by marked enlargement, dark discoloration, and rounded edges (Fig. 2C). The liver appeared congested and hemorrhagic (Fig. 2D and E). Tissue samples from the lungs, spleen, liver and kidney were collected for histopathological and molecular investigations. For histopathological analysis, the samples were fixed in 10% neutral buffered formalin for ten days. The fixed tissues were then dehydrated, cleared, and embedded in paraffin. Paraffin blocks were sectioned at a thickness of 4 μm and stained with hematoxylin and eosin (H&E) stains. Histopathological examination revealed widespread bronchitis (Fig. 3A) and pneumonia, with predominant proliferation of alveolar macrophages and other inflammatory cells within the peri-bronchial area and lung parenchyma (Fig. 3B). Additionally, there was an accumulation of proteinaceous exudates leading to alveolar collapse and damage, which was characterized by the loss of type I pneumocytes and proliferation of type II pneumocytes arranged in a palisading pattern (Fig. 3C). The spleen showed massive multifocal necrosis and lymphoid depletion, accompanied by thickened trabeculae (Fig. 3D). The liver exhibited widespread hepatocellular necrosis and portal hepatitis with predominant infiltration of macrophages. Additionally, intracytoplasmic inclusion bodies were observed within hepatocytes of the liver parenchyma (Fig. 3E and F).

**Fig. 4:**
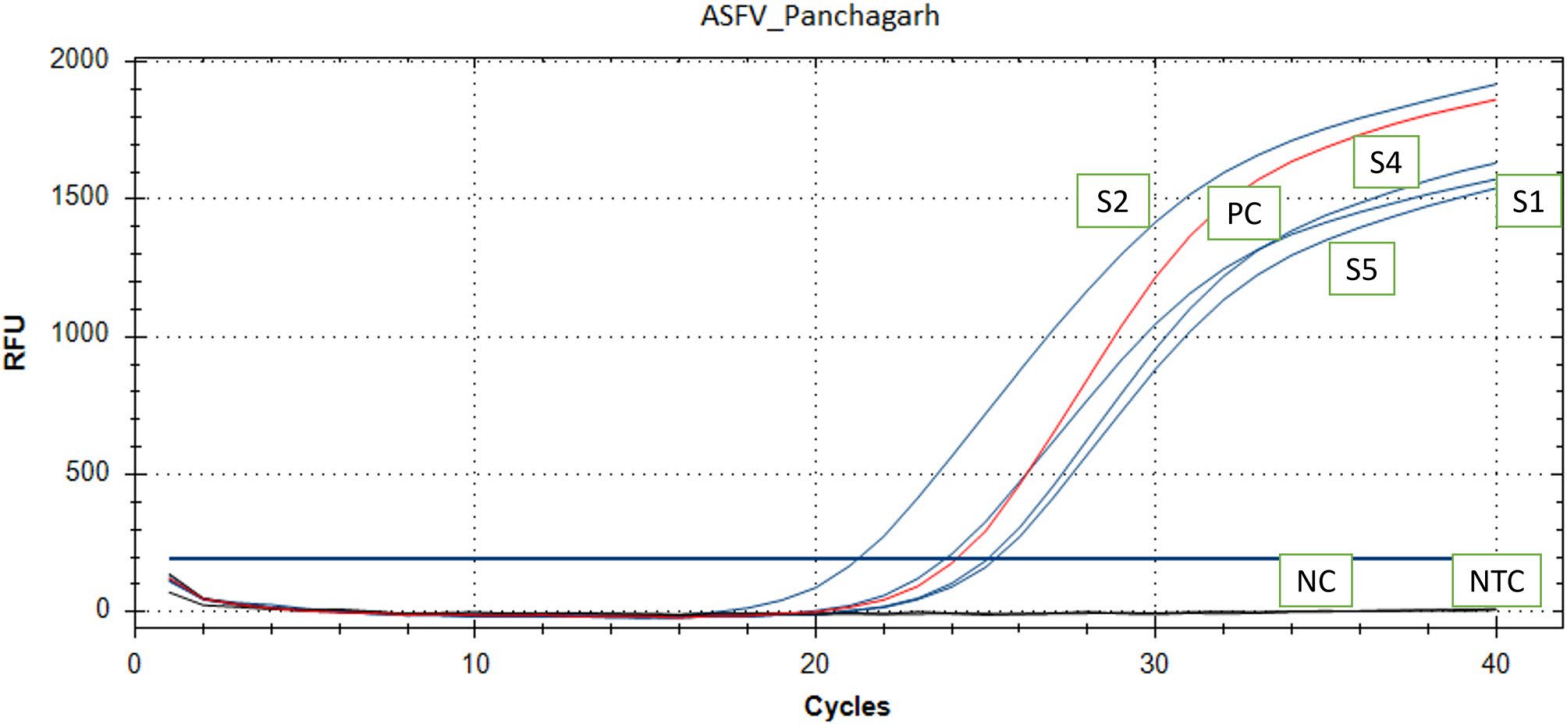
The qPCR amplification results showed the quantification cycle (Cq) values for the test samples, along with internal positive and negative controls to validate the assay

For molecular detection of ASFV, the lung, spleen, liver, and kidney tissues were excised from each of the four indigenous pigs. Equal volumes of each organ were pooled per animal and homogenized in a TissueLyser II system (Qiagen, Hilden, Germany) in phosphate-buffered saline (PBS) using a 1:10 (w/v) ratio. The homogenates were centrifuged at 6000 rpm for 5 minutes and the supernatants were collected. DNA from the clarified supernatants was then purified employing the DNeasy Blood and Tissue Kit for DNA isolation (Qiagen, Hilden, Germany) following the manufacturer’s instructions. Quantitative real-time PCR (qPCR) of the ASFV B646L gene of p72 protein was also carried out using previously validated primers and probes. Primers and probe used in the molecular detection were: 5’-CTGCTCATGGTATCAATCTTATCGA-3’ as forward, 5’-GATACCACAAGATC(AG)GCCGT-3’ and 5’-FAMCCACGGGAGGAATACCAACCCAGTG-3’-TAMRA as probe. Amplification reactions were carried out in the CFX90 real-time PCR thermal cycler (Bio-Rad, USA). Thermal cycling conditions were; initial denaturation at 50 °C for 2 min, denaturation at 95 °C for 10 min, and 40 cycles consisting of amplification with denaturation at 95 °C for 15 s and annealing/extension at 60 °C for 1 min. Fluorescence was measured after the extension step. Quantification cycle (Cq) values ≤ 30 were considered as positive.All the tissue samples tested had Cq values ranging between 21 and 25 (Fig. 4; Table 1), indicating positive for ASFV.

**Table 1:** The qPCR amplification results showed the quantification cycle (Cq) values for the test samples and internal positive control

Sample ID	Sample type	Cq value	Comments
S1	Pooled lungs, spleen, liver, and kidney tissues	24.16	Positive
S2	Pooled lungs, spleen, liver, and kidney tissues	21.58	Positive
S3	Pooled lungs, spleen, liver, and kidney tissues	25.60	Positive
S4	Pooled lungs, spleen, liver, and kidney tissues	25.35	Positive
PC	Internal positive control	24.44	Positive

## Discussion and conclusions

Although African swine fever (ASF) was previously reported in Bangladesh in 2023, earlier outbreaks lacked comprehensive clinical, pathological, and molecular investigations. The present study constitutes the first in-depth investigation of ASF affecting indigenous pigs in Bangladesh. Initial clinical findings observed included inappetence, high fever, staggering gait, and lack of fear, which ultimately progressed to death due to epistaxis and breathing difficulty. Similar clinical manifestations have been reported previously both in domestic and wild swine species [6, 11]. Additionally, the carcasses were frequently found near the water bodies, suggesting that the pigs may have sought cooler, moist environments in response to fever [11]. The mortality rate observed in this outbreak was 91.3%; however, mortality may reach up to 100% in both domestic and wild swine species, depending on the virulence of the virus strains [8].

Gross pathological examination revealed hallmark lesions of ASF, including severe pneumonia with red hepatization in different lobes, pulmonary edema, hemorrhagic splenomegaly, and hemorrhagic and congested liver. Similar observations have been documented in ASFV outbreaks in both Asia and Africa, reaffirming the aggressive nature of the virus and the challenges it poses for swine health and disease management. In a few previous studies, additional lesions such as petechial and ecchymotic hemorrhages in the heart, lymph nodes, kidneys, and intestines have been described [15]. However, such findings were not observed in the present study, which may be attributed to strain variation of the virus. Furthermore, while hemorrhages and cyanosis of the skin are commonly reported in domestic swine [15], these clinical signs were absent in the indigenous swine examined in this outbreak.

Histopathological analysis revealed widespread pneumonia in the lungs, massive multifocal necrosis in the spleen, and significant hepatocellular necrosis in the liver, accompanied by portal hepatitis and the presence of intracytoplasmic inclusion bodies. These observations are consistent with findings from previous studies [3, 6, 15]. Notably, there was a marked infiltration of alveolar macrophages in the lungs, as well as macrophage infiltration in the portal region and liver parenchyma, in line with earlier reports [12, 13]. Molecular confirmation of ASFV was achieved through a previously described qPCR assay targeting the B646L gene encoding the p72 protein [16]. To our knowledge, this field investigation represents the detailed pathological information of African swine fever in Bangladesh.

The lack of prior thorough investigations into ASF outbreaks in Bangladesh underscores the importance of the current study. Identifyingconsistent clinicopathological features and confirming viral presence through molecular detection may contribute to more effective surveillance, rapid diagnosis, and control strategies in future outbreaks. However, one of the primary limitations of this study was the inability to perform genetic characterization of the virus, which can help monitor viral strain introductions, genetic variations, and emerging epidemiological patterns of ASFV in Bangladesh.

Local farming practices, including backyard scavenging and swill feeding, are highly relevant to understanding ASF transmission dynamics in Bangladesh. As correctly noted, there are no structured or commercial pig farms in the country. Most pigs are raised under traditional, nomadic systems, often moving in flocks through different localities where they scavenge freely for food. Swill feeding is also likely to occur, though it is often informal and not well-documented by farmers. These practices create significant challenges for disease control and increase the risk of further spread of ASF. Furthermore, the detection of ASFV in indigenous swine populations raises concerns about potential endemic establishment and cross-border spread, especially given the limited veterinary infrastructure in some rural areas. These findings underscore the urgent need for targeted awareness programs, strengthened passive surveillance, and coordinated disease control efforts to prevent African Swine Fever from becoming endemic in South Asia.

## Data Availability

Data is provided within the manuscript.

## Abbreviations

ASF: African Swine Fever
ASFV: African Swine Fever Virus
qPCR: Real-time quantitative polymerase chain reaction
TAD: Transboundary animal disease
PIMs: Pulmonary intravascular macrophages
H&E: Hematoxylin and Eosin
